# Effect of Chitosan and Its Water-Soluble Derivatives on Antioxidant Activity

**DOI:** 10.3390/polym16070867

**Published:** 2024-03-22

**Authors:** Zhihua Wang, Yongbin Yan, Zhengmao Zhang, Changchun Li, Lanfei Mei, Ruyi Hou, Xiaodan Liu, Hongxia Jiang

**Affiliations:** 1 Hubei Key Laboratory of Quality Control of Characteristic Fruits and Vegetables, Hubei Engineering University, Xiaogan 432000, China; 2 School of Chemistry and Materials Science, Hubei Engineering University, Xiaogan 432000, China; 3 College of Fisheries, Henan Normal University, Xinxiang 453007, China

**Keywords:** chitosan, water-soluble derivatives, antioxidant activity, hyperlipidemia

## Abstract

The antioxidant activity of chitosan (CS) and three water-soluble derivatives was analyzed comparatively by in vitro and in vivo experiments, including hydroxypropyl chitosan (HPCS), quaternary ammonium salt of chitosan (HACC), and carboxymethyl chitosan (CMCS). The results show that chitosan and its water-soluble derivatives have a scavenging ability on DPPH radicals, superoxide radicals, and hydroxyl radicals, and a reducing ability. A remarkable difference (*p* < 0.05) was found for HACC and HPCS compared with CS on DPPH radicals, hydroxyl radicals, and reducing ability. The antioxidant ability of the four chitosan samples was in the order of HPCS > HACC > CMCS > CS. Furthermore, antioxidant activity of all samples increased gradually in a concentration-dependent manner. The in vivo result indicates that oral CS and its derivatives samples result in a decrease in lipid peroxides (LPO) and free fatty acids (FFA) levels in serum with an increase in superoxide dismutase (SOD) activity. Especially for the HPCS and HACC groups, the LPO, FFA, and SOD activity in serum was different significantly in comparison with the high-fat controlgroup (HF) (*p* < 0.05). These results indicate that chitosan and its derivatives can be used as good antioxidants, and the antioxidant activity might be related to the molecular structure of chitosan derivatives.

## 1. Introduction

Free radicals are generated during the normal metabolic process of human and animal bodies, and these include superoxide anion (•O_2_^−^), hydroxyl radicals (•OH), and hydrogen peroxide (H_2_O_2_), etc. Generally, the production and scavenging of free radicals maintains a balance within normal physiological free radical concentration in organisms. Excessive free radicals in the organism could lead to cell membrane destruction, protease inactivation, and gene damage and mutation, which could induce cancer, cardiovascular disease, diabetes, atherosclerosis, and other diseases [1,2]. In general, the administration of antioxidants could remove free radicals and active oxygen species in biological bodies, which is helpful for delaying or preventing the oxidation of intracellular oxidizable substrates, such as butylated hydroxytoluene, butylated hydroxy anisole, propyl gallate, and t-butylhydroquinone [3,4]. However, contrary to scavenging free radicals and protecting cells against free radical toxicity, these synthetic antioxidants have toxic effects on living organisms. There is growing interest in natural antioxidants from plants or other natural resources to replace synthetic antioxidants in medicine or food applications. Common natural antioxidants have scavenging effects on superoxide radicals, hydroxyl radicals, and lipid peroxides, and these include green tea, plant seeds, and vegetable extracts [5,6].

As a kind of biopolymer, chitosan has excellent biological activity, such as biodegradability, biocompatibility, antibacterial activity, antiviral activity, immune-enhancing and wound-healing activities, and so on [7,8]. At present, chitosan (CS) is widely applied as a polymer material in biomedicine and the food industry, including drug delivery systems and medical diets, wound dressings, and therapeutic supplements. Furthermore, chitosan could also be used in biosensors, radioprotectors, and so on [9]. However, the practical application of CS was restricted because of its poor solubility. In general, physical and chemical modification or its composite were needed to widen its application. For example, novel polymer therapeutics such as carboxymethyl chitosan-based nor cantharidin conjugates can be used against gastric cancer, graphene oxide/chitosan oligosaccharide/γ-polyglutamic acid composites can be used for anticancer drug delivery [10,11], and carboxymethyl chitosan can be used as a fruit juice clarificant [12]. A great deal of evidence shows that CS has antioxidant activity including scavenging free radicals and inhibiting ROS production in biological systems. Moreover, the antioxidant activity is affected by the degree of deacetylation, molecular weight (M_W_), and intramolecular/intermolecular hydrogen bonding, which is closely related to the active hydroxyl and amino groups of CS [13]. In order to improve the antioxidant activity of CS, it was of increasing interest to reduce its molecular weight under appropriate conditions. Chang et al. compared the in vitro antioxidant activity of CS before and after degradation, and the antioxidant effect of CS was higher with the decrease in molecular weight. The inhibitory activity of 2,2-diphenyl-1-picryl hydrazyl (DPPH) radicals for CS with 2.2 kDa had 80% more than that of with 300 kDa, whose radical scavenging activity was only 38.2% [14]. Tomida et al. achieved a similar result for the effect of molecular weight of CS on the scavenging effect of DPPH radicals and 2,2′-azinobis(3-ethylbenzothiazoline-6-sulfonic acid)(ABTS) [15]. In addition, Park et al. demonstrated that with the increase in deacetylation degree, the antioxidant activity of CS would increase [16]. Hromis et al. observed that the antioxidant activity of CS with 98% degree of deacetylation was 78.5%, while that of CS with 90% degree of deacetylation was only 51.5% [17].

In recent years, there have been attempts to improve the antioxidant activity of CS by chemical modification. The results show that chemically modification of CS by carboxymethyl, sulfate, and phenolic acid grafting could increase radical scavenging activity for CS derivatives [13,18,19]. After chemical modification, different chemical functional groups could bring about different effects on biological activities and physical properties. In this study, the antioxidant activities of three water-soluble chitosan derivatives, hydroxypropyl chitosan (HPCS), quaternary ammonium chitosan (HACC), and carboxymethyl chitosan (CMCS), were compared and analyzed from radical scavenging and reducing ability invitro and antioxidant activity in vivo. The relation between the antioxidant activity and the introduction of different substituent groups of CS was discussed.

## 2. Materials and Methods

### 2.1. Materials

Chitosan (M_W_, 1.5 × 10^5^; DD, 92%) was purchased from Golden-shell Biochemical Co., Ltd. (Yuhuan, China). All chemicals were obtained from Sinopharm Chemical Reagent Co., Ltd., Shanghai, China. All other biological reagents and assay kits of serum lipids were acquired from Nanjing Jiancheng Bioengineering Institute, China. 

### 2.2. Preparation and Characterization of Water-Soluble Chitosan Derivatives

Hydroxypropyl chitosan (HPCS) [20,21], chitosan quaternary ammonium salt (HACC) [22,23], and carboxymethyl chitosan (CMCS) [24,25] were prepared by etherification of CS with propylene oxide, hydroxypropyl trimethyl ammonium chloride, and chloroacetic acid under alkali conditions. In brief, chitosan was dispersed firstly in 33% alkali solution, and then frozenat −20 °C for 48 h to be activated. After thawing, the excess lye was squeezed out. Then the reaction continued for 8 h at 75 °C under N_2_ after adding excess reaction reagent. The obtained chitosan solution was precipitated in excess acetone, and the reaction products were dialyzed in deionized water to remove the excess reaction reagent and alkali. The water-soluble chitosan derivatives were obtained by freeze-drying. The structure of CS and its derivatives were analyzed using Fourier transform infrared spectrometer (FT-IR) with KBr pellets and ^1^H NMR. The substituting degree of HPCS, HACC, and CMCS were 0.79, 0.72, and 0.81, respectively.

### 2.3. Antioxidant Experiments

#### 2.3.1. DPPH Radical Scavenging Activity

The water-soluble derivatives of CS were prepared with double-distilled water, and CS was dissolved in acetic acid solution with different concentrations. 

The DPPH radical scavenging activity of CS and its derivatives was measured according to the method of Liu [18]. Briefly, 0.5 mL DPPH solution (0.1 mM, in 95% ethanol) was added to 2.5 mL four CS samples with various concentrations. The mixing solution was shaken well and incubated in the dark at room temperature for 90 min, and the absorbance was measured at 517 nm wavelength (*A*_2_). Sample solution was replaced by double-distilled water in blank group (*A*_0_), and DPPH solution was replaced by anhydrous ethanol in control group (*A*_1_). The radical scavenging activity of DPPH was calculated as follows:

$$\mathrm{DPPH}\;\mathrm{radical}\;\mathrm{scavenging}\;\mathrm{effect}/\%=(1-\frac{A_{2}-A_{1}}{A_{0}})\times 100$$

#### 2.3.2. Superoxide Radical Scavenging Activity

The superoxide radical scavenging activity was determined based on the method of Liu [18]. Reagent mixture, containing 50 μL CS samples, 4.5 mL 50 mmol/L pH 8.2 Tris-HCl buffer, and 25 μL 45 mmol/L pyrogallol solution was incubated at room temperature (25 °C) for 3 min, and then 1 mL 8 mmol/LHCl solution was dripped to terminate the reaction. Absorbance was read at 420 nm (*A*_2_). Sample solution was replaced by double-distilled water in the blank group (*A*_0_), and no pyrogallol was added in the control group (*A*_1_). The superoxide radical scavenging activity was analyzed using the following equation:

$$\mathrm{Superoxide}\;\mathrm{radical}\;\mathrm{scavenging}\;\mathrm{effect}/\%=(1-\frac{A_{2}-A_{1}}{A_{0}})\times 100$$

#### 2.3.3. Hydroxyl Radical Scavenging Activity

The hydroxyl radical scavenging activity was performed using the method of Zhong [26]. A mixture containing a 2.0 mL CS sample (of various concentrations), 2.0 mL 6 mmol/L FeSO_4_ solution, 2.0 mL 6 mmol/L H_2_O_2_ solution, and 2.0 mL 6 mmol/L salicylic acid was shaken vigorously and incubated at 37 °C for 30 min; absorbance was obtained at 510 nm wavelength (*A*_2_). In the blank group (*A*_0_) and control group (*A*_1_), double-distilled water was used to replace CS solution and salicylic acid solution, respectively. The hydroxyl radical scavenging rate was calculated by the following formula:

$$\mathrm{Hydroxyl}\;\mathrm{radical}\;\mathrm{scavenging}\;\mathrm{effect}/\%=(1-\frac{A_{2}-A_{1}}{A_{0}})\times 100$$

#### 2.3.4. Analysis of Reducing Power

The reducing power was analyzed referring to the method of Feng [27]. Reaction was performed in 0.5 mL CS sample solutions of various concentrations, mixed with 1.25 mL 0.05 mol/L sodium phosphate buffer (pH 6.6) and 1.25 mL K_3_Fe(CN)_6_(0.5%, *w*/*v*) by incubating in 50 °C water bath for 20 min. Afterwards, after adding 1.25 mL trichloroacetic acid (5%, *w*/*v*), the mixture was centrifuged at 4500 r/min for 15 min. 

The upper layer (3 mL) was mixed with 0.25 mL fresh FeCl_3_ (0.05%, *w*/*v*), and the reaction was kept at room temperature for 10 min. Absorbance was read at 700 nm wavelength (*A*_1_). In the blank group (*A*_0_), chitosan sample solution was replaced using double-distilled water. The reducing power was calculated by the following formula:

$$\mathrm{Reducing}\;\mathrm{power}/\%=\frac{A_{1}-A_{0}}{A_{0}}\times 100$$

### 2.4. Animal Experiments

Male Sprague-Dawley rats (120–140 g) with normal diets were supplied by Centers for Disease Control and Prevention of Hubei Province, China. All animal protocols were approved by the Institutional Animal Care and Use Committee of Hubei Experimental Animal Society. Ethical approval for the study was obtained from the local ethics committee at Hubei Engineering University.

All male rats were acclimatized with standard chow diets and water *ad libitum* in cases at room temperature for 7 days with 12 h light/dark cycle. On average, the rats were assigned into six groups randomly (8 rats in each group): normal control group (NC), high-fatgroup (HF), CS group, HPCS group, HACC group, and CMCS group. Firstly, NC group was fed with standard diets, and the other five groups all received high-fat diets containing 10% (*w*/*w*) egg yolk powder, 10% (*w*/*w*) lard, 0.2% (*w*/*w*) bile salt, 1.5% (*w*/*w*) cholesterol, and 78.3% basic diets. On the other hand, the NC and HF groups were administrated orally distilled water (1 mL/100 g) once daily, the CS group and three water-soluble derivatives groups were orally given 4.5% (*w*/*w*) CS dispersions and HPCS, HACC, CMCS solutions with the same dose, respectively. During the 30-day experiment, rats were allowed an *ad libitum* diet and water intake. Body weight (BW) and food intake were recorded every day. At last, after being fasted overnight, all rats were sacrificed. The serum was collected by centrifuging blood at 3000 rpm for 10 min at 4 °C. The serum samples were frozen and stored at −20 °C for biochemical analysis.

Serum triglyceride (TG), total cholesterol (TC), low-density lipoprotein cholesterol (LDL-C), high-density lipoprotein cholesterol (HDL-C), lipid peroxide (LPO), free fatty acid (FFA), and superoxide dismutase (SOD) levels were obtained from an assay of serum with commercial analytical kits (Nanjing Jiancheng Bioengineering Institute, Nanjing, China).

### 2.5. Statistical Analysis 

Values were expressed as mean ± SD. The Duncan test and one-way analysis of variance (ANOVA) were carried out, and the statistical analysis was enforced by the SPSS 22.0 software package. Statistical significance was accepted with *p* < 0.05.

## 3. Results

### 3.1. Structure Analysis of CS and Its Water-Soluble Derivatives

**Figure 1:**
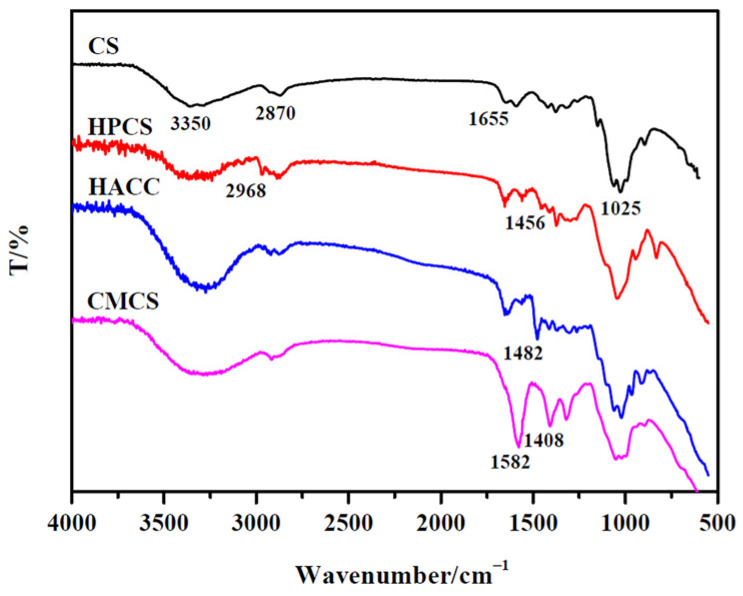
FT-IR spectra of CS and its water-soluble derivatives.

The IR spectra of CS and its derivatives are shown in Figure 1. From the IR spectrum of CS, a broad absorption band between 3600 and 3100 cm^−1^was assigned to N–H and O–H stretching vibrations. The peaks at 2870 cm^−1^ and 1025 cm^−1^were the characteristic absorption peaks of C–H and C–O–C, respectively. The band at 1655 cm^−1^represents a characteristic absorption peak of the acetylated amino group of CS, which indicates that the CS sample is not deacetylated fully. 

For HPCS, a stronger absorption band is shown at 1456 cm^−1^and a new absorption peak at 2968 cm^−1^, which are the joint contribution of the C–H asymmetry deformation and stretching vibration of –CH_3,_ respectively. This is closely related to the substituent hydroxypropyl group [20,21]. The IR spectrum of HACC exhibited a new absorption peak at 1482 cm^−1^, which was attributed to the bending vibration of the C–H of quaternary ammonium groups [22]. For CMCS, the symmetrical stretching vibration band of COOH at 1408 cm^−1^ occurred, but the asymmetric stretching vibration of COOH between 1720–1510 cm^−1^ was superimposed with the characteristic absorption band of acetylated amino group around 1655 cm^−1^, and a new strong absorption peak was shown at 1582 cm^−1^ [24,25]. However, compared with CS, the N–H bending of –NH_2_ around 1570 cm^−1^ was almost not changed for CS derivatives. These results reveal that the –OH groups of CS are mainly substituted under the experimental conditions.

**Figure 2:**
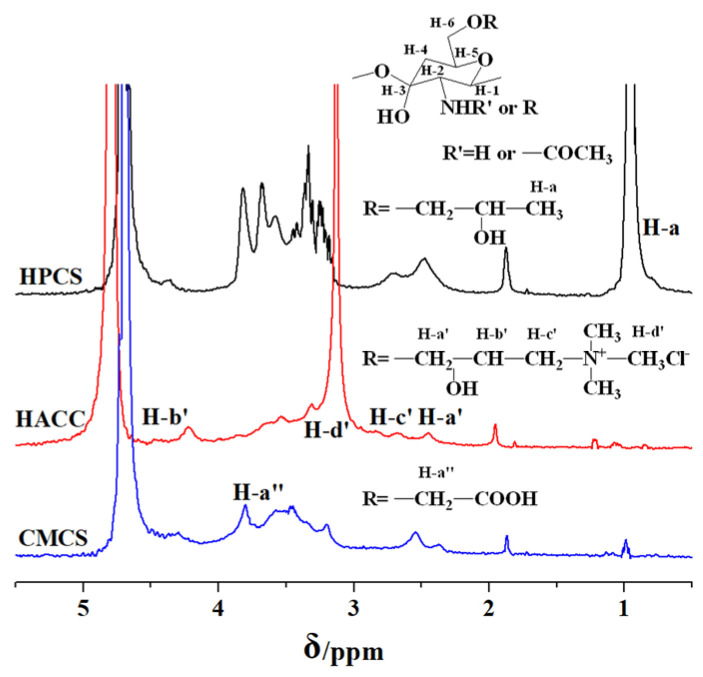
^1^H NMR spectra of CS and its water-soluble derivatives.

From the ^1^H NMR spectra of CS derivatives in Figure 2, the representative peaks for H-3, H-4, H-5, and H-6 of glucosamine rings appear between 3.1 ppm and 3.9 ppm, and the peaks of H-1 and H-2are shown at about 4.5 ppm and 2.5 ppm, respectively. For HPCS, the intensive signal is shown at 0.95 ppm, which is attributable to the characteristic peak of the proton of methyl group (H-a) of hydroxypropyl groups [21]. ^1^H NMR signals of HACC show that there was a most intensive peak at 3.12 ppm, which was associated with the methyl group (H-d′) of quaternary ammonium salt. Moreover, the representative peaks of the long quaternary ammonium salt segment (H-a′, H-b′, H-c′) are listed at 2.45 ppm, 4.47 ppm, and 2.84 ppm, respectively [22]. The spectrum for CMCS appeared with a peak at 3.81 ppm, which could be ascribed to the methyl group (H-a″) of carboxymethyl groups [24]. In addition, the representative peak of amine groups at about 1.9 ppm still existed after substitution.

### 3.2. DPPH Scavenging Activity

**Figure 3:**
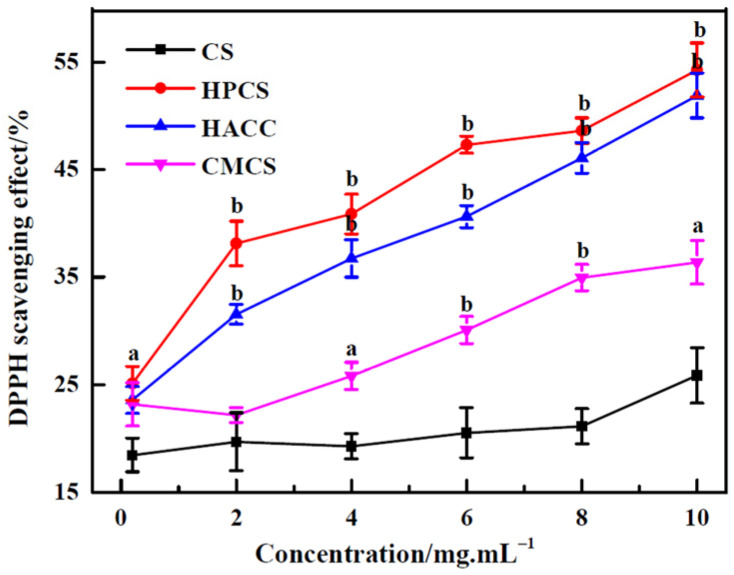
DPPH radical scavenging activity of chitosan and its derivatives. ^a^ *p* < 0.05, ^b^ *p* < 0.01, water-soluble derivatives vs. CS.

As shown in Figure 3, CS and its derivatives have different degrees of DPPH scavenging effects. The radical scavenging activity increased more or less with the increase in the concentration of all the samples. Compared with CS, the scavenging activities of HPCS, HACC, and CMCS were higher with a significant difference (*p* < 0.05). HPCS especially had a powerful scavenging ability on DPPH radicals, while CMCS was a poorer scavenger. Park et al. also indicated that the hydroxyl and amine groups of CS molecule were responsible for the radical scavenging activity [16]. The results imply that different functional groups of CS have certain effects on the DPPH scavenging activity.

### 3.3. Superoxide Radical (O_2_^−^) Scavenging Activity

Superoxide anion is a reduced form of molecular oxygen, which is generated through the metabolic process of a cell. It could interact with other molecules to produce secondary free radicals, such as hydroxyl radical, H_2_O_2,_ and singlet oxygen [28]. In general, pyrogallol was rapidly oxidized under alkaline conditions, which resulted in the release of superoxide anions, and, eventually, colored intermediate products were generated. In the present study, the scavenging activity assay of CS on the superoxide radicals was based on the inhibiting capacity of generating colored intermediate products.

**Figure 4:**
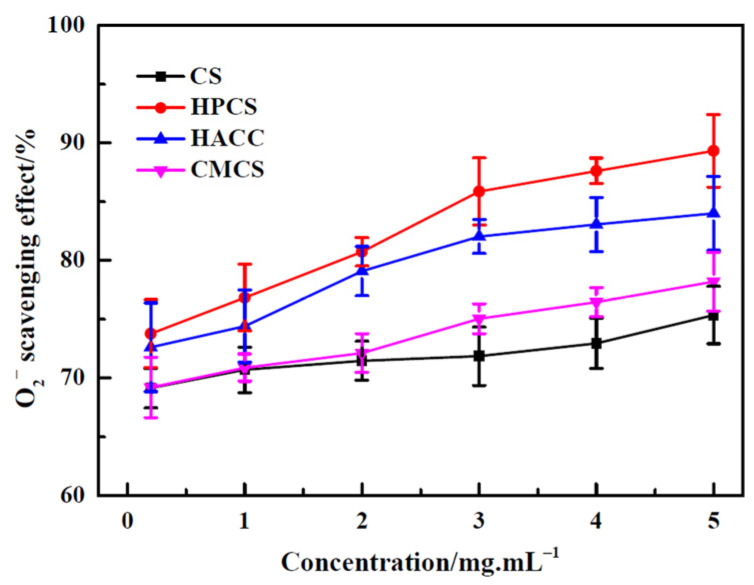
Superoxide radical scavenging activity of chitosan and its derivatives.

All the samples of CS and its derivatives had effective scavenging activity against superoxide radicals in Figure 4. The result shows that the scavenging activity of CS and its derivatives on superoxide radicals is in direct proportion to their concentration, which should be closely related to the activity of amino and hydroxyl groups of CS and its derivatives. The elimination effect of CS derivatives was higher slightly than that of CS on superoxide radicals, due to the fact that the structure of CS was severely disrupted by the introduction of functional groups after modification. Although there was no significant difference (*p* > 0.05), a good trend was still apparent.

### 3.4. Hydroxyl Radical (•OH) Scavenging Activity

**Figure 5:**
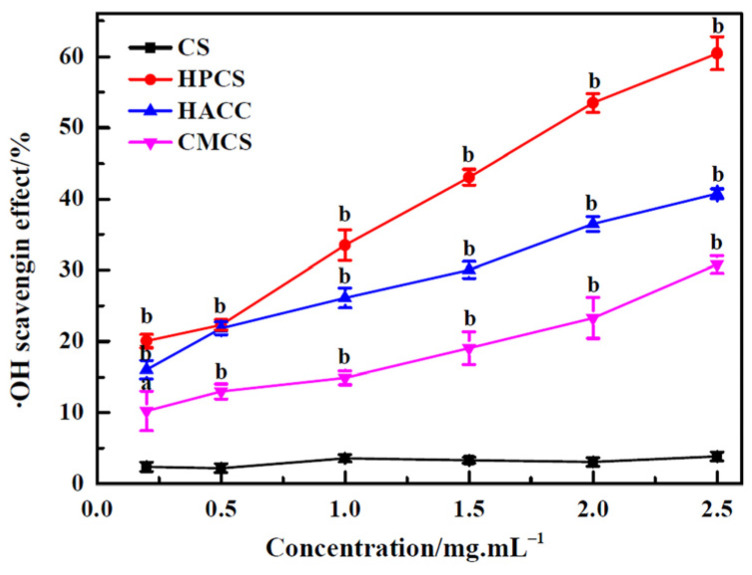
Hydroxyl radical scavenging activity of chitosan and its derivatives. ^a^ *p* < 0.05, ^b^ *p* < 0.01, water-soluble derivatives vs. CS.

For all ROS, hydroxyl radicals exhibited the strongest chemical activity, which could react easily with DNA, amino acids, and membrane components. In Figure 5, with the increase in CS concentration, there was no significant change in scavenging activity against hydroxyl radicals. Compared to CS, the three CS derivatives exhibited higher scavenging ability for hydroxyl radicals (*p* < 0.05), which might be attributed partially to their different metal chelating ability. The hydroxyl radical scavenging activity was the most prominent for HPCS. The Fe^2+^-chelating ability of CS was mainly in close relation with amino and hydroxyl groups, which contained lone pair electrons that helped to form chitosan–Fe^2+^complexes [27,29]. The stronger scavenging ability against hydroxyl radicals for HPCS may be due to the enhancement of Fe^2+^-chelating ability from the introduction of hydroxypropyl groups.

### 3.5. Reducing Power

**Figure 6:**
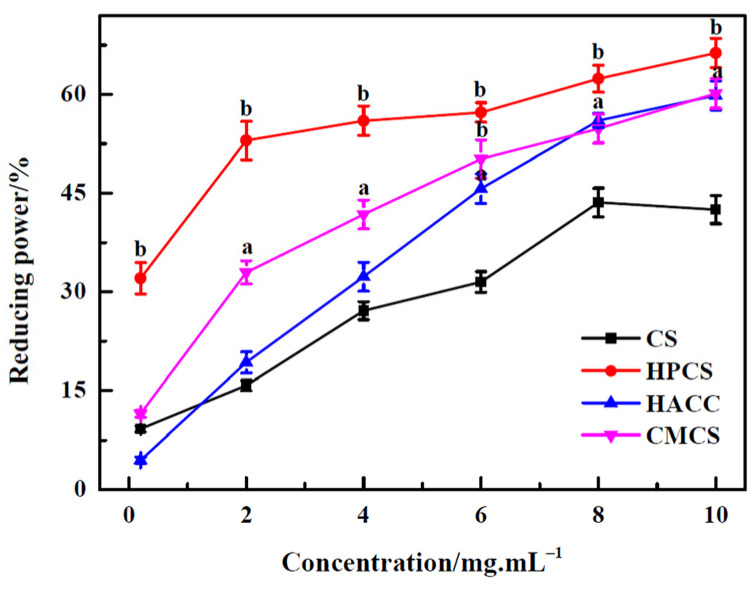
Reducing power of chitosan and its derivatives. ^a^ *p* < 0.05, ^b^ *p* < 0.01, water-soluble derivatives vs. CS.

The reducing capacity of the compounds is an important indicator of its potential antioxidant activity. The higher the reducing capacity of the compounds, the stronger its antioxidant activity. From Figure 6, it can be seen that the reducing power correlates well with the increase in all CS sample concentrations, but the reducing power of three CS derivatives is more prominent in comparison with CS. Especially for HPCS, there was significant difference (*p* < 0.01). The reducing property was generally related to the presence of a reductone to break the free radical chain by donating hydrogen atoms. It was also reported that the reductone could react with certain precursors of peroxide, and prevent peroxide formation [30]. This research result shows that the reducing power of CS is likely related to its hydrogen supply capacity. After chemical modification of CS, the activities of hydroxyl and amino groups and the substituent groups might affect the hydrogen donating ability of CS.

### 3.6. Analysis of the Rat Growth

BW, BW gain, and food intake for the rats is shown in Table 1. Comparing all groups, there was no significantly change in the daily food intake for each group of rats during the 30 day experiment. The general conditions of all rats were normal, but BW and BW gain of the HF group for high-fat-diet rats were significantly higher than that of the NC group (*p* < 0.05). Compared with the HF group, BW and BW gain of the CS group, HPCS group, HACC group, and CMCS group decreased. Especially for the two groups with HPCS and HACC intake, comparing with the HF group, there was significant difference in the final weight and BW gain (*p* < 0.05). 

### 3.7. Serum Lipid Analysis of the Rats

In order to investigate the effects of four chitosan samples on the serum lipid level, TG, TC, and LDL-C levels were measured after treatment with CS, HPCS, HACC, and CMCS for 30 days. As shown in Table 2, compared to the NC group, the levels of TG, TC, and LDL-C in the HF group are significantly higher, accompanied by the decrease in HDL-C level (*p* < 0.05), which suggests that a high-fat diet results in abnormal changes in elevated blood lipids in rats. After feeding chitosan and its derivatives, compared with the HF group, TG, TC, and LDL-C levels in rat serum decreased significantly (except TG for the CS group, *p* < 0.05), while serum HDL-C levels in the HPCS and HACC groups were higher. In conclusion, the regulating effect on serum lipid of CS and its derivatives was in the following order, HPCS > HACC > CMCS > CS. These results indicate that the four chitosan samples have a relatively good preventive effect on hyperlipidemia induced by high-fat diet in rats, and enhance their resistance to oxidative stress.

### 3.8. Oxidative Stress Analysis of the Rats

It was reported that high-fat diets could increase the generation of intracellular free radicals and induce tissue damage and that the control of free radicals was helpful for preventing hyperlipidemia. The oxidation state of biological organisms could be expressed using biochemical parameters, such as FFA, LPO, and SOD activity. As shown in Table 3, compared with the control group, serum LPO and FFA of the HF group increased prominently (*p* < 0.01), and serum SOD activity decreased prominently (*p* < 0.01). This indicates that high-fat diets aggravate the oxidative stress of rats and cause the accumulation of lipid metabolism and oxidation products. Nevertheless, the oral CS and its derivatives could help to increase SOD activity (except the CS group), and decrease LPO and FFA levels in serum in comparison with the HF group. Particularly, the biological parameters of the HACC and HPCS groups show significant differences compared to the HF group (*p* < 0.05, except for LPO of the HACC group). These results indicate that CS and its derivatives have a role in alleviating oxidative stress effects.

## 4. Discussion

As a natural polymer, CS has excellent biological activity including antioxidant activity. But the antioxidant mechanism was always controversial. In theory, the hydroxyl and amino groups (–OH and –NH_2_) of chitosan molecules should have radical scavenging activity. But some studies indicate that native CS has low or no antioxidant activity due to the intra and intermolecular hydrogen bonds [13,31]. It turned out that the antioxidant activity of CS increased significantly after being modified [32]. In this research, the antioxidant ability was analyzed by comparing the scavenging and reducing ability of CS and three derivatives, HPCS, HACC, and CMCS, on DPPH radicals, superoxide radicals, and hydroxyl radicals in vitro, as well as the antioxidant activity in vivo for hyperlipidemia rats. The overall results show that the antioxidant activity of four chitosan samples increased proportionally with the increase in their concentrations, but their antioxidant effects were significantly different as a result of the change in functional groups of CS macromolecules.

The strong intra and intermolecular hydrogen bond of CS affects the activity of -OH and –NH_2_. After chitosan was dissolved in acidic media, its –OH and –NH_2_ groups still showed some activity. As reported by Marin et al., CS had remarkable metal chelate ability stemming from chelate ligands of –NH_2_ and –OH groups [33]. Xie et al. proved that the amino groups of CS could react with free radicals to form stable macromolecular free radicals [34]. Although the N-atom of the CS molecule could donate an unshared pair of electrons for antioxidant ability, the radical scavenging ability and reducing ability of –OH and –NH_2_ were still weakened due to the protonation of –NH_2_ under the acidic conditions ofthe CS solution [32]. 

After carboxymethyl modification, CMCS had good solubility and showed a slightly better radical scavenging effect compared with CS [24]. As reported, the hydroxyl content of CS affects the scavenging activities of hydroxyl radicals and superoxide anion [25], because the hydroxyl groups were substituted partly by carboxymethyl groups, and also for CMCS, the radical scavenging ability was still weak. Compared with CMCS, it was found that HACC exhibited a more significant effect on the radical scavenging and reducing ability in this study. It had been proven that quaternary CS has better scavenging activity on hydroxyl radicals than a CS Schiff base [35]. This is closely related to the different forms of nitrogen atom in CS molecules, such as primary amine, secondary amine, tertiary amine, and quaternary ammonium. After being quaternized, the positive charge density of the N-atom at C-2 in HACC molecules was strengthened, and the antioxidant capacity was improved [4]. Moreover, our result also indicates that HPCS has a significantly higher radical scavenging ability and reducing power than HACC in vitro, which is closely related to the introduction of hydroxypropyl groups onto the CS molecular chain. Liu et al. found that more hydroxyl groups on the aromatic ring were beneficial to improve the antioxidant activity. The antioxidant ability of caffeic-acid-grafted CS was higher in comparison with ferulic-acid-grafted CS, because there were two substituted hydroxyl groups on the para-position and ortho-position of the aromatic ring of caffeic acid [18]. 

Lipid peroxidation was considered to be the result of oxidative stress, which originated from the disruption of the dynamic equilibrium between the antioxidant and prooxidant mechanisms [36]. Based on the above in vitro experimental data, further study onantioxidant properties of the four dietarychitosan samples was carried out in vivo from several core biological parameters. The result shows that high-fat diet caused the lipid accumulation and strengthened the oxidative stress of rats from the change in serum lipids, LPO levels, serum FFA, and the SOD activity for the high-fat diet group in comparison with the NC group (Table 2 and Table 3). But feeding CS and water-soluble derivatives not only decreased serum lipid significantly, but also enhanced the resistance to oxidative stress during the 30-day period for the rats with a high-fat diet. The relevant study also proved that chitosan and its derivatives had a good lipid-lowering effect [37]. Moreover, the serum levels of TG, TC, and LDL-C decreased markedly for hyperlipidemic rats after being fed with CS and water-soluble chito-oligosaccharides [38]. 

This suggests that CS and its derivatives could be used as protein antioxidants to reduce oxidative stress for renal failure [39]. Furthermore, rats being fed media-milled chitosan was helpful to decrease the levels of FFA and TBARS and increase SOD activity [40]. In this study, it was confirmed that the order of CS and its three derivatives on lipid-lowering activity and the resistance to oxidative stress in vivo were as follows: HPCS > HACC > CMCS > CS. It might be due to the better solubility of the three water-soluble derivatives, which contributed to inhibiting the lipid absorption and decreasing lipid concentration more effectively in the small intestine in comparison with CS. In addition, the surface positive charge of HACC made it easy to bind to negatively charged lipids, and its lipid-lowering effect was better than that of negatively charged CMCS. It was reported that oral O-carboxymethyl chitosan and N-[(2-hydroxy-3-N, N-dimethylcetylammonium) propyl] chitosan could help to reduce serum TG and FFA for high-fat-dietrats, and their lipid-lowering activity was superior to that of CS [37]. Furthermore, the hydroxyl content of CS derivatives affected its antioxidant activity. Compared with CS, the hydroxyl group content of HPCS was basically unchanged, while carboxymethyl and quaternary ammonium groups replaced some hydroxyl groups of CS, resulting in the decrease in hydroxyl group content of CMCS and HACC. HPCS also had the highest antioxidant activity in vivo.

## 5. Conclusions

All in all, antioxidant analysis shows that chitosan and its derivatives could decrease free radical levels in a concentration-dependent manner in vitro, and different substituent groups affected the antioxidant activity of CS. Meanwhile, in vivo experiments show that the four chitosan samples could reduce the serum lipid level for hyperlipidemia rats, and increase the activity of SOD.

The research findings suggest that HACC and HPCS were more significant than CMCS and CS on the antioxidant defense system of living organisms against the free radicals and the regulation of lipid accumulation. Therefore, chitosan derivatives could be used as natural good antioxidants, which opens up a new path for the development of natural pollution-free biological antioxidants.

## Figures and Tables

**Table 1:** Body weight parameters of rats at the end of 30 day experiment (g $\bar{x}$ ± s, n = 8).

Group	Initial Weight (g)	Final Weight (g)	BW Gain (g)	Food Intake (g/d)
NC	169.4 ± 11.7	324.5 ± 13.4	155.1 ± 11.2	19.9 ± 2.1
HF	171.2 ± 12.5	381.5 ± 15.5 *	210.3 ±14.3 *	20.7 ± 2.9
CS	170.1 ± 12.6	344.0 ±14.6 ^a^	173.9 ±11.2	21.1± 1.9
HPCS	176.4 ±11.8	333.2 ± 15.2 ^a^	156.8 ±12.0 ^a^	20.9 ± 3.3
HACC	173.1 ± 10.6	329.1 ± 16.4 ^b^	156.0 ±11.9 ^a^	20.1 ± 1.5
CMCS	172.2 ±11.3	356.6 ± 16.9 *	181.1 ±13.5	19.7± 2.8

* *p* < 0.05 vs. NC group; ^a^ *p* < 0.05, ^b^ *p* < 0.01 vs. HF group.

**Table 2:** Blood lipid change after treatment with four chitosan samples in rats ($\bar{x}$ ± s, n = 8).

Group	TG (mmol/L)	TC (mmol/L)	HDL-C(mmol/L)	LDL-C (mmol/L)
NC	0.37 ± 0.06	2.65 ± 0.71	0.89 ± 0.16	1.47 ± 0.45
HF	0.85 ± 0.14 **	7.77 ± 0.67 **	0.79 ± 0.28	6.68 ± 0.77 **
CS	0.64 ± 0.09 **	6.11 ± 0.90 **^a^	0.65± 0.11 *	4.08 ± 0.59 **^b^
HPCS	0.34 ± 0.04 ^b^	3.45 ±0.81 *^b^	0.86 ± 0.17	3.34± 0.73 **^b^
HACC	0.35 ± 0.06 ^b^	4.65 ±0.59 **^b^	0.85 ± 0.21	4.06 ± 0.83 **^b^
CMCS	0.40 ± 0.07 ^b^	6.32 ± 0.38 **^a^	0.72 ± 0.18	3.51 ± 0.62 **^b^

* *p* < 0.05, ** *p* < 0.01 vs. NC group; ^a^ *p* < 0.05, ^b^ *p* < 0.01 vs. HF group.

**Table 3:** Antioxidant status of four chitosan samples in vivo ($\bar{x}$ ± s, n = 8).

Group	FFA (μmol/L)	LPO (μmol/L)	SOD (U/mL)
NC	158.1 ± 25.3	5.64 ± 0.74	366.8 ± 37.9
HF	758.5 ± 48.5 **	18.05 ± 3.69 **	255.6 ± 16.4 **
CS	742.8 ± 66.4 **	17.33 ±4.34 **	230.3 ± 13.4 **
HPCS	269.6 ± 25.4 *^b^	7.89 ±1.36 **^b^	314.7 ± 19.2 ^a^
HACC	495.7 ± 65.6 **^b^	12.98 ±1.79 **	280.4 ± 37.9 **^a^
CMCS	689.4 ± 77.0 **^a^	17.65 ± 1.64 **	265.5 ± 35.6 **

* *p* < 0.05, ** *p* < 0.01 vs. NC group; ^a^ *p* < 0.05, ^b^ *p* < 0.01 vs. HF group.

## Data Availability

The data presented in this study are available on request from the corresponding author.
